# Anaerobic utilization of Fe(III)‐xenosiderophores among *Bacteroides* species and the distinct assimilation of Fe(III)‐ferrichrome by *Bacteroides fragilis* within the genus

**DOI:** 10.1002/mbo3.479

**Published:** 2017-04-11

**Authors:** Edson R. Rocha, Anna S. Krykunivsky

**Affiliations:** ^1^ Department of Microbiology and Immunology Brody School of Medicine East Carolina University Greenville NC; ^2^ Intern from the Undergraduate Research Internship Placement Program University of the West of England (UWE) Bristol UK

**Keywords:** Anaerobes, anaerobic bacteria, bacteroides, iron, xenosiderophores

## Abstract

In this study, we show that *Bacteroides* species utilize Fe(III)‐xenosiderophores as the only source of exogenous iron to support growth under iron‐limiting conditions in vitro anaerobically. *Bacteroides fragilis* was the only species able to utilize Fe(III)‐ferrichrome while *Bacteroides vulgatus* ATCC 8482 and *Bacteroides thetaiotaomicron* VPI 5482 were able to utilize both Fe(III)‐enterobactin and Fe(III)‐salmochelin S4 as the only source of iron in a dose‐dependent manner. We have investigated the way *B. fragilis* assimilates Fe(III)‐ferrichrome as initial model to understand the utilization of xenosiderophores in anaerobes. *B. fragilis* contains two outer membrane TonB‐dependent transporters (TBDTs), FchA1 and FchA2, which are homologues to *Escherichia coli* ferrichrome transporter FhuA. The disruption of *fchA1* gene had only partial growth defect on Fe(III)‐ferrichrome while the *fchA2* mutant had no growth defect compared to the parent strain. The genetic complementation of *fchA1* gene restored growth to parent strain levels indicating that it plays a role in Fe(III)‐ferrichrome assimilation though we cannot rule out some functional overlap in transport systems as *B. fragilis* contains abundant TBDTs whose functions are yet not understood. However, the growth of *B. fragilis* on Fe(III)‐ferrichrome was abolished in a *feoAB* mutant indicating that Fe(III)‐ferrichrome transported into the periplasmic space was reduced in the periplasm releasing ferrous iron prior to transport through the FeoAB transport system. Moreover, the release of iron from the ferrichrome may be linked to the thiol redox system as the *trxB* deletion mutant was also unable to grow in the presence of Fe(III)‐ferrichrome. The genetic complementation of *feoAB* and *trxB* mutants completely restored growth on Fe(III)‐ferrichrome. Taken together, these findings show that *Bacteroides* species have developed mechanisms to utilize ferric iron bound to xenosiderophores under anaerobic growth conditions though the regulation and role in the biology of *Bacteroides* in the anaerobic intestinal environment remain to be understood.

## 1 Introduction

The human colon is the most densely populated organ with commensal microbes and *Bacteroides* species are among the predominant members of that microbiota (Eckburg et al., 2005; Gibson & Roberfroid, 1999; Hooper, Midtvedt, & Gordon, 2002; Savage, 1977). Colonization by *Bacteroides* spp. is fundamental for the establishment and maintenance of a normal, healthy intestinal microbiota and disruption of this commensal relationship has a great impact on health and disease. In the human colon, *Bacteroides* spp. can reach numbers in excess of 10^11^ cells/g of feces and account for about 30–40% of total bacteria where at least 500 different species have been so far reported (Hooper et al., 2002; Smith, Rocha, & Paster, 2006; Xu & Gordon, 2003; Xu et al., 2003). The contribution of this predominant group of bacteria in the large intestine is related to a variety of physiological functions. As an example, *Bacteroides* spp. are involved directly in complex polysaccharide degradation, bile acid turnover metabolism, proteolytic activity, transformation of toxic and mutagenic compounds, regulation of host fat storage, development of the immune system and protection against pathogens (Eckburg et al., 2005; Jarchum & Pamer, 2011; Neish, 2009; Neu, Douglas‐Escobar, & Lopez, 2007; Reading & Kasper, 2011; Savage, 1977; Smith et al., 2006; Tappenden & Deutsch, 2007).

The diverse bacterial population within the human colon makes this environment a highly competitive ecosystem and in order for *Bacteroides* spp. to maintain their high cell number, they need to compete efficiently for the available nutrients with other components of the microflora (Fuller & Perdigón, 2003). Among the essential nutrients required by *Bacteroides spp*. are iron and heme. *Bacteroides* spp. have an essential requirement for heme and nonheme‐iron and growth can be stimulated in a dose‐dependent manner by heme (Rocha, de Uzeda, & Brock, 1991; Rocha & Smith, 2010; Sperry, Appleman, & Wilkins, 1977; Varel & Bryant, 1974). The *Bacteroides* are not able to synthesize the tetrapyrrole protoporphyrin IX but can synthesize heme if protoporphyrin IX and a source of inorganic iron is provided in vitro (Rocha & Smith, 2010; Rocha et al., 1991; Sperry et al., 1977). However, there is a paucity of information regarding how *Bacteroides* species respond to and acquire iron in the anaerobic environment of the human colon. Iron has a remarkable influence on the gut microbiota. The competition for iron fluctuates the balance among commensal bacteria, and iron limitation prevents the colonization of pathogens and mucosa inflammation (Buhnik‐Rosenblau, Moshe‐Belizowski, Danin‐Poleg, & Meyron‐Holtz, 2012; Deriu et al., 2013; Dostal et al., 2012;, Jaeggi et al., 2015; Krebs et al., 2013; Werner et al., 2011; Zimmermann et al., 2010).

Early studies using Enterobacteria as a model have demonstrated that ferrous iron rather than ferric iron was the most important form of iron available to enteric bacteria in the anaerobic environment of the lower intestinal tract (Hantke, 2004; Stojiljkovic, Cobeljic, & Hantke, 1993; Tsolis, Bäumler, Heffron, & Stojiljkovic, 1996). However, recent studies have shown that acquisition of ferric iron via siderophores plays a fundamental role in facultative bacteria colonization of the murine intestinal tract (Pi et al., 2012). In the intestinal tract, ferric iron may be present as insoluble precipitated forms of phytates, carbonates, phosphates, and tannates, and by autooxidation of ferrous iron adjacent to oxygenated mucosal surface (Babbs, 1992; Conrad & Umbreit, 2000). The presence of ferric iron in the colon correlates with recent studies demonstrating that *E. coli* mono‐ or dual‐associated with *Bacteroides thetaiotaomicron* in the colonic mucus layer of germ‐free mice induces the expression of genes required for synthesis and uptake of catechol‐type siderophore enterobactin as well as for the uptake of the hydroxamate‐type ferrichrome for the acquisition of ferric iron (Li et al., 2015). These studies indicate that both ferrous and ferric forms of iron are present in the colon but their availability is likely to be limited (Kortman, Raffatellu, Swinkels, & Tjalsma, 2014). Siderophores are low molecular high‐affinity iron chelators synthesized by many microorganisms to forage insoluble ferric iron in aerobic environments or from host tissues iron‐binding proteins when iron availability is limiting (Chu et al., 2010; Ratledge & Dover, 2000).

Aerobic and Facultative Gram‐negative bacteria utilize specific outer membrane TonB‐dependent transporters (TBDTs) to transport iron‐chelates across the outer membrane and into the periplasmic space where periplasmic‐binding proteins and membrane ATP‐binding transporters facilitate their translocation into the cell (Faraldo‐Gómez & Sansom, 2003; Noinaj, Guillier, Barnard, & Buchanan, 2010; Schalk, Mislin, & Brillet, 2012). Transport of substrates through TBDT is energy‐dependent which is derived from the proton motive force and transduced to the outer membrane transporter by the integral inner membrane complex TonB/ExbB/ExbD (Noinaj et al., 2010; Schalk et al., 2012; Schauer, Rodionov, & de Reuse, 2008). Gram‐negative bacteria induce synthesis of TBDTs in response to iron limitation to transport Fe(III)‐siderophores produced by themselves or by other organisms (xenosiderophores) (Armstrong, Brickman, & Suhadolc, 2012; Chu et al., 2010; Galet et al., 2015; Guan, Kanoh, & Kamino, 2001; Joshi, Archana, & Desai, 2006; Krewulak & Vogel, 2008, 2011; Noinaj et al., 2010; Ratledge & Dover, 2000; Strange, Zola, & Cornelissen, 2011; Tanabe et al., 2012). Bacteria also utilize cell‐signaling ECF sigma/antisigma and two‐component regulatory systems to induce the expression of cognate TBDTs in response to the presence of xenosiderophores they are designed to transport (Gasser et al., 2016; Llamas et al., 2006, 2008).

*Bacteroides* species do not appear to produce known siderophores (Otto, Verweij‐van Vught, van Doorn, & Maclaren, 1988; Rocha et al., 1991) yet they do co‐exist in a habitat densely populated with organisms known to produce siderophores. Therefore, it is reasonable to speculate that *Bacteroides* could take advantage of xenosiderophores to acquire iron for growth. The *Bacteroides* robust nutritional versatility is highlighted by the presence of nearly one hundred TBDTs in their genomes which is more than any other bacterium (Cerdeño‐Tárraga et al., 2005; Koebnik, 2005; Patrick et al., 2010; Schauer et al., 2008; Xu et al., 2003). The majority of *Bacteroides* TBDTs are utilized to import complex polysaccharides and host glycans important for energy generation (Martens, Kelly, Tauzin, & Brumer, 2014; Martens et al., 2011), but for many of these TBDTs receptors the specific substrates and nutritional role remain unknown. Thus in this study, we show that *Bacteroides* have the capability to grow in the presence of Fe(III)‐xenosiderophores under iron‐limiting conditions anaerobically in vitro. We also show that there is differential assimilation of iron bound to hydroxamate and catechol type siderophores among major *Bacteroides* species that colonize the human colon. The growth stimulation of *B. fragilis* by Fe(III)‐ferrichrome and of *B. vulgatus* and B. *thetaiotaomicron* by Fe(III)‐enterobactin and Fe(III)‐salmochelin S4 indicates that *Bacteroides* species have developed significant differences in the way they acquire and compete for iron in the intestinal ecological system.

## 2 Materials and methods

### 2.1 Strains and growth conditions

*Bacteroides* strains and plasmids used in this study are shown in Table 1. Strains were routinely grown anaerobically in brain heart infusion broth supplemented with 5 μg/ml hemin, 1 g/L‐cysteine, and NaHCO_3_ (BHIS). Rifamycin (20 μg/ml), 100 μg/ml gentamicin, 5 μg/ml tetracycline, and 10 μg/ml erythromycin were added to the media when required. For growth dependence on Fe(III)‐siderophore, a modified semidefined medium (SDM) (Rocha & Smith, 2004) was used as follow: KH_2_PO_4_, 1.5 g/L; NH_4_SO_4_, 0.5 g/L; NaCl, 0.9 g/L; L‐methionine, 150 mg/L; vitamin B12, 5 μg/L; MgCl_2_.6H_2_O, 20 mg/L; CaCl_2_.2H_2_O, 10 mg/L; MnCl_2_.4H_2_O, 1 mg/L; CoCl_2_.6H_2_O, 1 mg/L; resazurin, 1 mg/L; L‐cysteine, 1 g/L; protoporphyrin IX, 5 mg/L; glucose, 5 g/L; tryptone, 1 g/L. Twenty ml of 10% NaHCO_3_ were added per liter of medium, final pH 7.2. For some experiments, heme was omitted and replaced with protoporphyrin IX (PpIX) as source of tetrapyrrole macrocycle (Rocha et al., 1991). For iron restriction in SDM, the ferrous iron chelator bathophenanthroline disulfonic acid (BPS), which does not enter the cell (Alcaín, Löw, & Crane, 1995; Hassett, Romeo, & Kosman, 1998) was added at 20 μmol/L final concentration. Ammonium ferrous sulfate (Sigma‐Aldrich) at 200 μmol/L was added for ferrous iron‐replete growth conditions. The iron‐free siderophores, ferrichrome (Sigma‐Aldrich), ferrioxamine E (Sigma‐Aldrich), and deferrioxamine (Sigma‐Aldrich) were dissolved in 0.85% sodium chloride and filtered sterilized on 0.20 μm cellulose membrane (Corning Inc., Corning NY). Enterobactin iron‐free (Sigma‐Aldrich), salmochelin S4 iron‐free (Genaxxon bioscience, Germany) and pyoverdine iron‐free (Sigma‐Aldrich) were dissolved in dimethyl sulfoxide (DMSO) and filtered sterilized as above. Iron‐free siderophore stock solutions at 2 mmol/L were mixed with sterile 1 mmol/L ammonium Fe(III) citrate (Sigma‐Aldrich) in distilled water at 1:1 (v/v) overnight to obtain 1 mmol/L siderophore solution with 50% iron‐saturation containing the chelated iron at 0.5 mmol/L in the Fe(III)‐siderophore complexed form. Inoculum cultures were prepared by inoculating 8–10 colonies from fresh cultures grown for 24–48 hr on BHIS plates into 3 ml of SDM broth plus PpIX with addition of 10 μmol/L BPS and incubated anaerobically at 37°C for approximately 24 hr or until reaching OD_550 nm_ of 1.0 allowing exhaustion of cellular endogenous iron. Fresh SDM PpIX media containing 20 μmol/L BPS was inoculated with 1:50 inoculum dilution and supplemented with 0.5 mmol/L Fe(III)‐siderophore solution to final concentrations indicated in the text. For bioassay on BHIS plates, hemin was replaced with 10 μg/ml PpIX and supplemented with 1 mmol/L BPS. A 6 mm sterile disk paper filter was placed on top of inoculated plates and two times 10 μl of 0.5 mmol/L Fe(III)‐siderophore solution was applied on the disk. After 24 hr at 37°C anaerobic incubation, additional two times 10 μl were applied and incubated for 5–6 days.

**Table 1:** *Bacteroides* strains and plasmids used in this study

Strains	Relevant genotype	References
*B. fragilis* 638R	Clinical isolate, Rif	Privitera, Dublanchet, & Sebald, 1979
*B. fragilis* NCTC 9343	Abdominal infection	NCTC
*B. fragilis* CLA 267	Clinical isolate Tet Cfx	P. C. Applebaun^a^
*B. fragilis* IB370	638R *trxB::cfxA Rif Cfx*	Rocha, Tzianabos, & Smith, 2007
*B. fragilis* IB383	638R *trxB::cfxA* pFD892/*trxB* ^*+*^ *Erm*	Rocha et al., 2007
*B. fragilis* BER‐51	638R *ΔfeoAB::tetQ*, Rif Tet	Veeranagouda et al., 2014
*B. fragilis* BER‐120	638R *fchA2*::pFD516 Rif Erm	This study
*B. fragilis* BER‐125	BER‐51 pER‐191Tet Erm	Veeranagouda et al., 2014
*B. fragilis* BER‐127	638R *fchA1*::pYT102 Rif Tet	This study
*B. fragilis* BER‐128	BER‐127 *fchA2*::pFD516 Rif Erm Tet	This study
*B. fragilis* BER‐130	BER‐127 carrying pER‐201 Erm	This study
*B. fragilis* BER‐131	BER‐128 carrying pER‐201 Erm	This study
*B. ovatus* ATCC 8483		ATCC
*B. thetaiotaomicron* VPI 5482		VPI
*B. vulgatus* ATCC 8482		ATCC
*B. vulgatus* ATCC 29327		ATCC
*B. vulgatus* CLA 341		P. C. Applebaun^a^
*B. vulgatus* 20‐15	Human patient with ulcerative colitis isolate	Onderdonk, Steeves, Cisneros, & Bronson, 1984
*B. vulgatus* 40G2‐33	Guinea pig with cecal ulceration isolate	Onderdonk et al., 1984
*B. vulgatus* 10‐9	Health human fecal isolate	Onderdonk et al., 1984
*B. vulgatus* 16‐4	Health human fecal isolate	Onderdonk, Bronson, & Cisneros, 1987
Plasmids		
pYT102	*Bacteroides* suicide vector, Cm, Tet	Baughn & Malamy, 2002
pFD340	*Bacteroides* expression shuttle vector, Amp, Erm	Smith, Rogers, & McKee, 1992
pFD516	*Bacteroides* suicide vector, Sp, Erm	Smith, Rollins, & Parker, 1995
pER‐186	A 0.715 bp BamHI/SstI internal N‐terminus of *fchA2* was cloned into the BamHI/SstI sites of pFD516	This study
pER‐178	An approximately 2.4 kb BamHI/EcoRI fragment from pFD340 was deleted and replaced with an approximately 2.4 kb BamHI/EcoRI *cfxA* gene. Amp Cfx	This study
pER‐194	A 0.604 kb Bamhi/HindIII internal DNA fragment of *fchA1* was cloned into the BamHI/HindII sites of pYT102	This study
pER‐201	A 2,522 bp BglII/BamHI promoterless *fchA1* DNA fragment was cloned into the BamHI site of pER‐178.	This study

Erm, erythromycin resistance; Cfx, cefoxitine resistance; Rif, rifamycin resistance; Tet, tetracycline resistance; Cm, chloramphenicol resistance; ATCC, American Type Culture Collection; NCTC, National Collection of Type Cultures; VPI, Virginia Polytechnic Institute and State University.

a Strain provided by P. C. Applebaun, Department of Pathology, Hershey Medical Center, Pennsylvania 17033.

### 2.2 Construction of *B. fragilis fchA1* (BF638R_0018) and *fchA2* (BF638R_2503) insertional mutants

An internal 608 nt DNA fragment encompassing nt 64 through 672 of the BF638R_0018 gene locus was PCR amplified using primers Bf‐0018‐BamHI‐Forward (GCCACGGATCCAGAGTCTGTCG) AND Bf‐0018‐HindIII‐Reverse (CTGTAAGCTTTCTACTCCCTGC). The amplified fragment was digested with BamHI/HindIII and cloned into the BamHI/HindIII sites of the *E. coli*‐*Bacteroides* shuttle suicide vector pYT102 (Baughn & Malamy, 2002). The new construct, pER‐194, was mobilized from *E. coli* DH10B into *B. fragilis* 638R by triparental filter mating protocols previously described (Shoemaker, Getty, Gardner, & Salyers, 1986). Transconjugants were selected on BHIS agar containing 20 μg of rifamycin per ml, 100 μg of gentamicin per ml and 5 μg of tetracycline per ml. PCR amplification analysis was used to confirm single cross‐over insertion of pER‐194 into the new strain BER‐127 (*fchA1::*pYT102).

An internal 715 nt DNA fragment encompassing nt number 14 through the 729 of the BF638R_2503 ORF was PCR amplified using primers Bf‐2503‐BamHI‐Forward (GAAAAGGATCCTATTAGCTGC) and Bf‐2503‐SstI‐Reverse (CGCGGTGAGCTCCGATACGG). The amplified fragment was digested with BamHI/SstI and cloned into the BamHI/SstI sites of the *E. coli*‐*Bacteroides* shuttle suicide vector pFD516 (Smith et al., 1995). The new construct, pER‐186, was mobilized from *E. coli* DH10B into *B. fragilis* 638R by triparental filter mating protocols previously described. Transconjugants were selected on BHIS agar containing 20 μg of rifamycin per ml, 100 μg of gentamicin per ml and 10 μg of erythromycin per ml. PCR amplification analysis was used to confirm single cross‐over insertion of pER‐186 into the new strain BER‐120 (*fchA2::*pFD516).

The construction *B. fragilis fchA1 fchA2* double mutant strain (BER‐128) was obtained by mobilizing pER‐194 from *E. coli* DH10B into BER‐120 strain by triparental mating as described above. Transconjugants were selected on BHIS agar containing 20 μg/ml of rifamycin, 100 μg/ml gentamicin, 10 μg/ml erythromycin and 5 μg/ml tetracycline.

### 2.3 Genetic complementation

For genetic complementation of BER‐127 and BER‐128 strains, a 2,522 nt promoterless DNA fragment of the BF638R_0018 gene locus (*fchA1*) containing 47 nt upstream the ATG codon was PCR amplified using primers Bf‐0018‐BglII_comp‐Forward (GGTACACAGATCTTTGCGGCTCGC) and Bf‐0018‐BamHI_comp‐Reverse (GCTGATCAGGATCCCTGCCGG) and cloned into the BamHI site of the modified pFD340 (Smith et al., 1992) *Bacteroides* expression vector pER‐178. The new construct, pER‐201, was conjugated into BER‐127 and BER‐128 by triparental mating to obtain BER‐130 and BER‐131 strains respectively.

## 3 Results

### 3.1 Growth stimulation of *Bacteroides* species by Fe(III)‐siderophores

**Figure 1:**
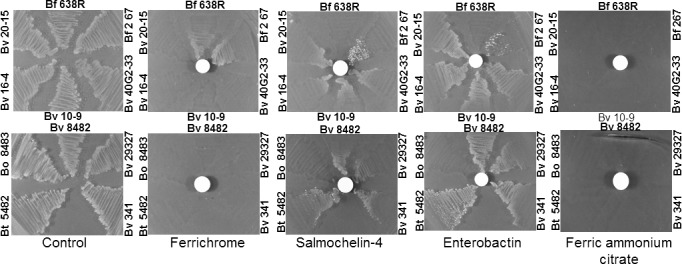
Fe(III)‐siderophore‐dependent growth of *Bacteroides* species on BHIS plates containing 10 μg/ml protoporphyrin IX and 1 mmol/L bathophenanthroline disulfonic acid. Siderophores used are depicted below respective panel. Fe(III)‐siderophore solutions at 0.5 mmol/L were applied onto paper disks placed on top of plates according to the procedures described in the material and methods section. Bacterial colonies grown around the paper disks indicated that growth was stimulated by the addition of respective Fe(III)‐siderophore. Growth control plates contain10 μg/ml protoporphyrin IX plux 200 μmol/L ammonium ferrous sulfate. Abbreviations of the bacterial strains and designations used are labeled beside respective inoculum region in each panel as follow: Bf 638R: *B. fragilis* 638R, Bf 267: *B. fragilis* CLA 267, Bo 8483: *B. ovatus* ATCC 8483, Bt 5482: *B. thetaiotaomicron* VPI 5482, Bv 8482: *B. vulgatus* ATCC 8482, Bv 29327: *B. vulgatus* ATCC 29327, Bv 10‐9: *B. vulgatus* 10–9, Bv 16‐4: *B. vulgatus* 16–4, Bv 20‐15: *B*. *vulgatus* 20–15, Bv 341: *B. vulgatus* CLA 341, Bv 40G2‐33: *B. vulgatus* 40G2‐33

Fe(III)‐bound siderophores are able to stimulate and support growth of *Bacteroides* species as the only available form of exogenous iron anaerobically when PpIX was used as the source of tetrapyrrole macrocycle. The *B. fragilis* 638R, NCTC 9343 and CLA 267 strains were able to grow on solid media around the filter disk loaded with the hydroxamate Fe(III)‐ferrichrome as the only source of iron. None of the other *Bacteroides* species tested were able to grow on Fe(III)‐ferrichrome (Figure 1 and Table S1). Interestingly, the *B. vulgatus* and *B*. *thetaiotaomicron* strains grew on the catecholates Fe(III)‐enterobactin and Fe(III)‐salmochelin S4 but not on Fe(III)‐ferrichrome (Figure 1 and Table S1). The *B. fragilis* CLA 267 also grew in the presence of both Fe(III)‐enterobactin and Fe(III)‐salmochelin S4. *B. ovatus* ATCC 8483 did not grow in the presence of ferrichrome, enterobactin or salmochelin S4. Moreover, none of the *Bacteroides* strains tested grew in the presence of Fe(III)‐ferrioxamine E, Fe(III)‐pyoverdine, Fe(III)‐deferrioxamine (Table S1) or Fe(III)‐dihydroxybenzoic acid (data not shown). Taken together, these findings indicate that there are significant differences in the assimilation systems of iron‐bound xenosiderophore among intestinal *Bacteroides* species. No growth was observed around the control plates containing filter disks loaded with ammonium ferric citrate indicating that only iron‐loaded siderophores were able to promote bacterial growth.

### 3.2 Growth on Fe(III)‐ferrichrome is not totally dependent on the presence of the TonB‐dependent outer membrane proteins FchA1 (BF638R_0018) and FchA2 (BF638R_2503)

As shown in Figure 1, *B. fragilis* strains were the only *Bacteroides* species to utilize Fe(III)‐ferrichrome for growth under iron‐limiting conditions on solid agar media. This suggests that a ferrichrome transport system is likely to be present in *B. fragilis* but absent in other *Bacteroides* species. This prompted us to characterize the role of putative TBDTs in assimilation of Fe(III)‐ferrichrome.

There are approximately 98 outer membrane TBDTs annotated in the genome of *B. fragilis* 638R of which 33 are predicted to be TBDTs containing 22 strand antiparallel β‐barrel signature of siderophore transporters and 67 belonging to the SusC family of nutrient transporters ((http://www.ncbi.nlm.nih.gov/nuccore/FQ312004.1; Cerdeño‐Tárraga et al., 2005). A BLAST search revealed two TBDT genes BF638R_0018 and BF638R_2503, henceforth named *fchA1* and *fchA2* respectively, that are each highly conserved (97%–100% amino acid identity) among all *B. fragilis* genomes available in the GenBank (83 genomes) but absent in nearly all other *Bacteroides* species genomes including the *B. vulgatus* ATCC 8482, *B. thetaiotaomicron* VPI 5482 and *B. ovatus* ATCC 8483 strains. These findings correlate with the bioassay results above showing that only *B. fragilis* was able to grow on Fe(III)‐ferrichrome. FchA1 and FchA2 have amino acid sequence homologies (23% identity, 42% similarity and 24% identity, 41% similarity, respectively) to the well‐studied *E. coli* Fe(III)‐ferrichrome transporter FhuA (Locher et al., 1998). FchA1 and FchA2 only share 27% identity and 36.5% similarity to each other suggesting that there are significant differences in transport functions and substrate specificities. In fact, an alignment of the amino acid sequence of *E. coli* FhuA with FchA1 and FchA2 homologs shows that FchA1 but not FchA2 contains a C‐terminal phenylalanine residue important for assembly into the outer membrane (de Cock, Struyvé, Kleerebezem, van der Krift, & Tommassen, 1997; Struyvé, Moons, & Tommassen, 1991). Moreover, the ferrichrome‐binding amino acid residues of *E. coli* FhuA plug region (R81, G99 and Y116) and barrel region (Y244 and Y315) (Locher et al., 1998) are not conserved in either FchA1 or FchA2 (Figure S1). An unrooted phylogenetic analysis of TBDT homologs in *B. fragilis* 638R showed that both FchA1 and FchA2 are related to *E. coli* FhuA in a distinct branch (Figure S2).

**Figure 2:**
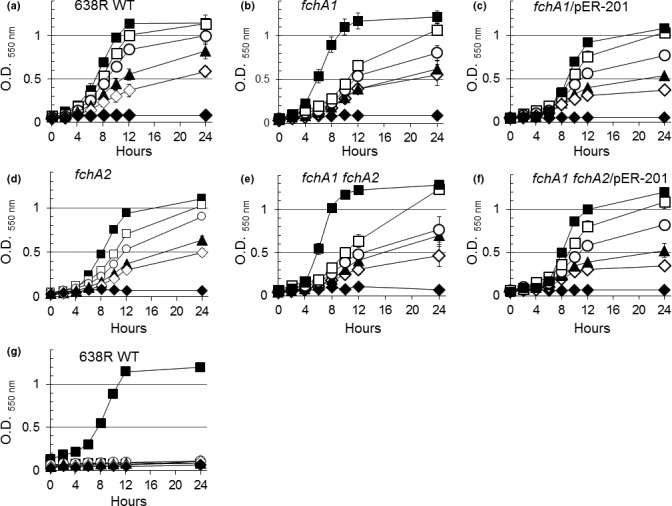
Growth of *B. fragilis* mutant strains in the presence of Fe(III)‐ferrichrome (a–f) and ammonium Fe(III) citrate (g). Strain designations are depicted in each panel. Bacteria were grown on SDM containing 5 μg/ml protoporphyrin IX and 20 μmol/L bathophenanthroline disulfonic acid. Fe(III)‐ferrichrome (Panels a–f) or ammonium Fe(III) citrate (Panel g) were added at the following final concentrations: No addition (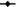), 0.1 μmol/L (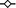), 0.5 μmol/L (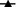), 2 μmol/L (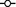), and 5 μmol/L (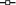). Ammonium ferrous sulfate at 200 μmol/L (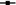) was added for iron‐replete growth controls in all panels. Data presented are an average of two determinations in duplicate (a–f) and one determination in duplicate (g). Vertical bars represent standard deviation. SDM, semidefined medium

These findings prompted us to investigate whether FchA1 and FchA2 would affect growth in the presence of Fe(III)‐ferrichrome. The parent strain, *B. fragilis* 638R, grew on Fe(III)‐ferrichrome on a dose‐dependent manner while addition of iron in the form of ammonium ferric citrate did not stimulate growth under iron‐limiting conditions (Figures 2a,g). The growth of an *fchA1* mutant (BER‐127) and the *fchA1 fchA2* double mutant (BER‐128) was partially attenuated when grown on 2 μmol/L and 5 μmol/L Fe(III)‐ferrichrome compared to the parent strain (Figure 2b,e). In contrast, the growth of *fchA2* single mutant (BER‐120) was not significantly altered compared to parent strain (Figure 2d). The growth rates of *fchA1* or *fchA2* mutants were not affected at low concentrations of Fe(III)‐ferrichrome though the genetic complementation of BER‐127 and BER‐128 with the *fchA1* gene (BER‐130 and BER‐131 respectively) restored the growth deficiency at 5 μmol/L Fe(III)‐ferrichrome (Figure 2c, f). This suggests that FchA1 is only partly involved in Fe(III)‐ferrichrome utilization. Moreover, the expression of *fchA1* and *fchA2* mRNAs was not regulated by either inorganic iron‐ or heme‐limiting conditions (Table S2). Taken together, the findings suggest that iron homeostasis is not responsible for control of *fchA1* and *fchA2* in the uptake and transport of Fe(III)‐ferrichrome in *B. fragilis*. Despite our limitations in identifying such transporters, we believe that Fe(III)‐ferrichrome utilization in *B. fragilis* is an active mechanism, and not an artifact effect of growth, because Fe(III)‐ferrichrome has no growth stimulation effect on neither of the related species *B. vulgatus, B. thetaiotaomicron* nor *B. ovatus* under the same growth conditions (Figure 1 and 4c–d).

### 3.3 The ferrous iron transporter *feoAB* is required for growth on Fe(III)‐ferrichrome

**Figure 3:**
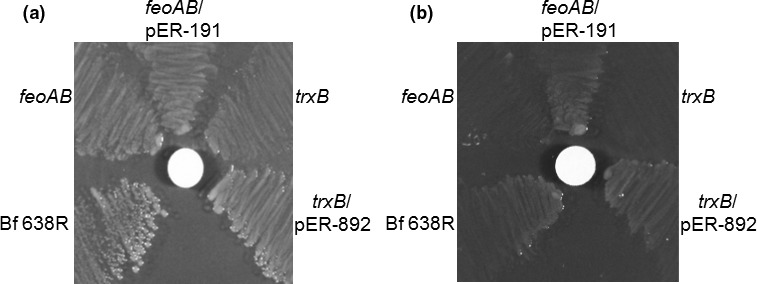
Growth deficiency of *B. fragilis feoAB* and *trxB* mutant strains on Fe(III)‐ferrichrome. (a) BHIS plate containing 5 μg/ml protoporphyrin IX plus 200 μmol/L ammonium ferrous sulfate for bacteria growth control. (b) BHIS plates containing 5 μg/ml protoporphyrin IX plus 1 mmol/L bathophenanthroline disulfonic acid as ferrous chelator for exogenous free iron‐limiting conditions. In panel a, sterile saline was added as control of the solvent on the paper disk. In panel b, Fe(III)‐ferrichrome at 0.5 mmol/L solution was added on the paper disk as described in the materials and methods section. Strains designation are labeled in each panel

Interestingly, *B. fragilis* does not contain homologs of the well‐characterized FhuCDB and FhuF systems of *E. coli* necessary for binding Fe(III)‐ferrichrome in the periplasmic space, transport across the cytoplasmic membrane, and then reduce it to release ferrous iron in the cytoplasm (Cooper, McArdle, & Raymond, 1978; Fischer, Strehlow, Hartz, & Braun, 1990; Mademidis et al., 1997; Matzanke, Anemüller, Schünemann, Trautwein, & Hantke, 2004). This indicates that assimilation of Fe(III)‐ferrichrome in *B. fragilis* may differ from the classical mechanism described for facultative Gram‐negative bacteria. We hypothesized that the reduction and release of iron from the Fe(III)‐ferrichrome complex would occur in the periplasm of *B. fragilis* and the free ferrous iron would be transported into the cytoplasm by the ferrous iron transporter hybrid component system FeoAB (Veeranagouda et al., 2014). To test this, we used the *feoAB* deletion mutant strain to determine whether it would have growth deficiency in the presence of Fe(III)‐ferrichrome as the only source of exogenous iron. In fact, the *feoAB* mutant no longer grows on the agar plate in the presence of Fe(III)‐ferrichrome (Figure 3). The genetic complementation of the *feoAB* with wild‐type gene completely restored the ability of the BER‐51 strain to grow on Fe(III)‐ferrichrome. These findings support our hypothesis that iron released from Fe(III)‐ferrichrome in the periplasmic space is transported into the cytoplasm through the FeoAB system.

The mechanism(s) responsible for the reductase activity that causes reduction of ferric iron and its dissociation from ferrichrome in the periplasmic space under anaerobic conditions is not yet known. Nevertheless to investigate whether the redox thiol/disulfate homeostasis in *B. fragilis* would affect growth on Fe(III)‐ferrichrome, the thioredoxin reductase (TrxB) deletion mutant strain was used (Rocha et al., 2007). Indeed, the *trxB* mutant was unable to grow around the disk filter containing Fe(III)‐ferrichrome (Figure 3). The genetic complementation of the *trxB* mutant with wild‐type *trxB* gene, strain IB383, restored growth on the bioassay plate similar to the parent strain growth (Figure 3). These results clearly indicate that normal physiological redox control is required for this anaerobe to utilize exogenous iron in the form of Fe(III)‐ferrichrome.

### 3.4 Fe(III)‐enterobactin and Fe(III)‐salmochelin S4 support growth of *B. vulgatus* ATCC 8482

**Figure 4:**
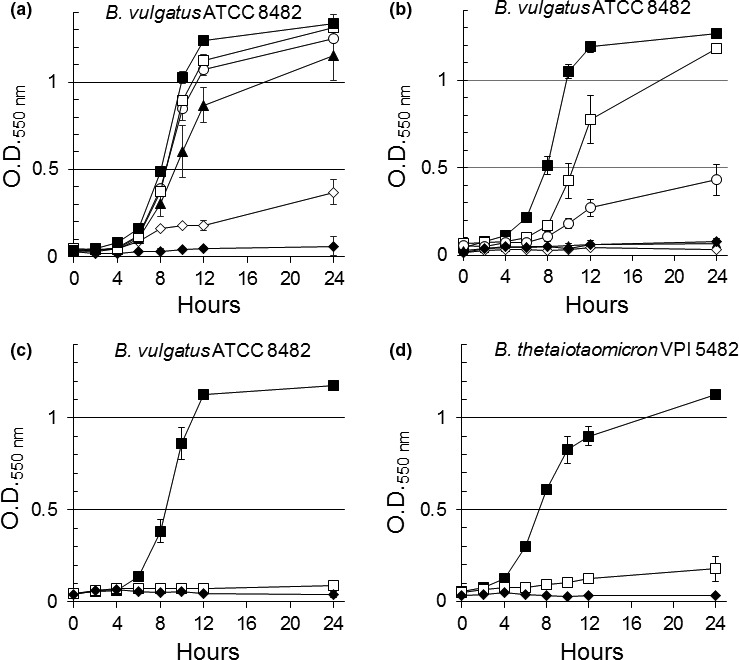
Growth of *B. vulgatus* ATCC 8482 (a, b, and c) and *B. thetaiotaomicron* VPI‐5482 (d) on Fe(III)‐siderophores. (a) Fe(III)‐enterobactin. (b) Fe(III)‐salmochelin S4. (c–d) Fe(III)‐ferrichrome. Bacteria were grown on SDM media containing 5 μg/ml protoporphyrin IX and 20 μmol/L bathophenanthroline disulfonic acid. Fe(III)‐siderophores were added at the following concentrations: No addition (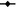), 0.1 μmol/L (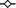), 0.5 μmol/L (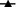), 2 μmol/L (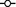), 5 μmol/L (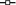). Ammonium ferrous sulfate at 200 μmol/L (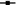) was added for iron‐replete growth controls. Panels c and d show the growth on Fe(III)‐ferrichrome at 5 μmol/L only for clarity. Data presented are an average of two determinations in duplicate. Vertical bars represent standard deviation. SDM, semidefined medium

When *B. vulgatus* ATCC 8482 was cultured in SDM PpIX under iron‐limiting conditions, it grew in the presence of Fe(III)‐enterobactin in a dose‐dependent manner from 0.1 μmol/L to 5 μmol/L. Nearly optimal maximum growth was obtained with 0.5 μmol/L Fe(III)‐enterobactin compared to growth in iron‐replete media (Figure 4a). In contrast, no significant growth occurred when salmochelin S4 was used at 0.1 μmol/L or 0.5 μmol/L. Partial growth occurred at 2 μmol/L while at 5 μmol/L there was a significant growth stimulation reaching maximum growth levels after 24 h compared to iron‐replete conditions (Figure 4b). These findings indicate that Fe(III)‐enterobactin seems to be more efficient in promoting growth of *B. vulgatus* at lower concentrations than does salmochelin S4 (Figure 4a ,b).

**Figure 5:**
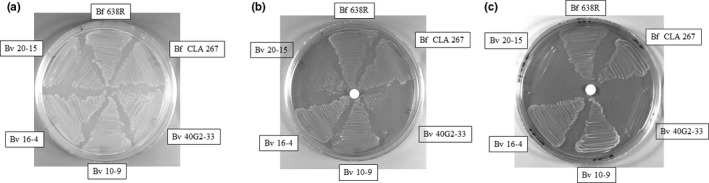
Growth of *B. vulgatus* (Bv) and *B. fragilis* (Bf) strains on BHIS plates supplemented with 100 μg/ml hemin plus (a) 200 μmol/L ammonium ferrous sulfate or b and c) 1 mmol/L bathophenanthroline disulfonic acid. (b) Fe(III)‐enterobactin was added onto the filter disk paper as described in the material and methods section. (c) A solution of 50% DMSO in distilled water was added as onto the disk paper as solvent control. Bacteria strains designation are depicted in each panel

The importance of Fe(III)‐enterobactin assimilation in *B. vulgatus* was further demonstrated for the colitis‐associated *B. vulgatus* 40G2‐33 and 20‐15 strains in the presence of heme (Figure 5). Interestingly, the *B. vulgatus* 40G2‐33 and 20–15 strains are unable to grow in the presence of heme at concentrations up to100 μg/ml as the sole source of iron (Figure 5c). However, growth of *B. vulgatus* 40G2‐33 and 20‐15 can occur in the presence of heme if Fe(III)‐enterobactin (Figure 5b) or inorganic iron are provided exogenously (Figure 5a). In contrast, the control strains *B. vulgatus* 10–9 and 16–4 isolated from healthy individuals and *B. fragilis* grew on heme alone as expected (Figure 5c) since in the absence of exogenous iron, iron can be obtained from heme (Rocha et al., 1991; Sperry et al., 1977; Verweij‐Van Vught, Otto, Namavar, Sparrius, & Maclaren, 1988). It is important to mention that growth of *Bacteroides* species is not stimulated in media lacking heme or protoporhyrin IX (Rocha et al., 1991; Sperry et al., 1977; Verweij‐Van Vught et al., 1988). Taken together, these findings clearly show that intestinal *Bacteroides* species have developed different strategies to acquire heme‐iron and Fe(III)‐siderophores for growth under iron‐limiting conditions anaerobically.

## 4 Discussion

In this study, we have demonstrated that *Bacteroides* species have developed strategies for acquisition of Fe(III)‐bound siderophores produced by other organisms in vitro. The distinct utilization of the hydroxamate Fe(III)‐ferrichrome by *B. fragilis* and the catecholates enterobactin and salmochelin S4 by *B. vulgatus and B. thetaiotaomicron* is an advantage for competition for iron and it is likely that it may play a role in growth and composition of the intestinal microflora. The utilization of xenosiderophores by *Bacteroides* spp. may not only enable them to obtain iron for their own metabolism but also to scavenge intestinal iron as a advantage against competing organisms by limiting environmental iron resources. These findings add further support to reports that commensal microflora play a role in protecting the host against intestinal colonization by pathogenic bacteria by competing and disrupting their ability to forage iron in the lower intestinal tract (Ellermann & Arthur, 2016; Kortman et al., 2014). Very little is known about the mechanisms by which *Bacteroides* acquire iron in the intestinal tract. We have analyzed the ability of *B. fragilis* to assimilate iron bound to ferrichrome as an initial model system to understand how anaerobic organisms have developed mechanisms to acquire and utilize ferric iron‐bound siderophores which is a hallmark of iron utilization by aerobic and facultative organisms.

Although progress has been made in recent years in the understanding of the structures and functions of the SusC‐like protein family of TBDTs (Foley, Cockburn, & Koropatkin, 2016; Martens et al., 2011, 2014), very little is known about *Bacteroides* TBDTs role in the assimilation of iron‐chelate complexes. *Bacteroides* species contain an extensive number of predicted TBDTs potentially involved in iron acquisition but their substrates and regulatory controls have not been well defined compared to the classical TBDTs in aerobic and facultative bacteria whose cognate substrates and regulation of the transport mechanisms are well understood (Koebnik, 2005; Schalk & Guillon, 2013; Schalk et al., 2012; Schauer et al., 2008). In this study, our efforts to determine the role of FchA1 and FchA2 revealed that only FchA1 plays a role in supporting growth on ferrichrome while FchA2 did not affect growth. In *E. coli*, deletion of *fhuA* completely impaired the transport of and growth on Fe(III)‐ferrichrome (Carmel, Hellstern, Henning, & Coulton, 1990). Therefore, we speculate that in view of the abundant number of TBDTs in *B. fragilis*, it is likely that redundancy in affinity transport of Fe(III)‐ferrichrome is present and highlight the possibility that transport of chelated iron diverges from the eubacterial models.

Another difference between iron utilization in *B. fragilis* and *E. coli* is the absence of the periplasmic Fe(III)‐ferrichrome‐binding protein FhuD and of the ATP‐binding cassette transporter FhuBC. In the cytoplasm of *E. coli*, ferric iron‐bound hydroxamate is released via reduction to ferrous iron with the involvement of the ferric reductase FhuF (Cooper et al., 1978; Matzanke et al., 2004). Mutants defective in FhuE were significantly impaired in their ability to remove iron from coprogen, ferrichrome and ferrioxamine B (Matzanke et al., 2004). In contrast to facultative bacteria, our findings suggest that Fe(III)‐ferrichrome is reduced in the periplasmic space to release free ferrous iron because the inner membrane ferrous iron transporter *feoAB* mutant has a growth defect when Fe(III)‐ferrichrome is used as the sole source of iron. In this regard, the *B. fragilis* FeoAB system which is regulated by iron limitation in a classical Fur‐dependent manner (Veeranagouda et al., 2014) may be a major controller of the way *B. fragilis* regulates the levels of iron that enters cytoplasm to maintain intracellular inorganic iron homeostasis. In support of this, our preliminary studies suggest that this is the case for the removal of iron from heme which also occurs extra‐cytoplasmically and the assimilation of heme‐iron for growth is dependent on the presence of the FeoAB system (unpublished data). This mechanism involving reduction and release of iron from siderophore in the bacterial periplasm has also been shown to occur in *Pseudomonas aeruginosa* (Greenwald et al., 2007; Marshall, Stintzi, Gilmour, Meyer, & Poole, 2009). Moreover, in the case of ferric iron released from citrate as ferrous iron in the periplasm, it requires the presence of FeoB for transport into the cell (Marshall et al., 2009).

Though *B. fragilis* has a reducing periplasmic space (Dutton, Boyd, Berkmen, & Beckwith, 2008; Shouldice et al., 2010; Tang, Dallas, & Malamy, 1999), the pathway for ferric iron reductase activities required for Fe(III)‐ferrichrome reduction is unclear. Recent studies have shown that the *B. fragilis* periplasmic thioredoxin (TrxP) contributes through cycles of reduction and oxidation activities to maintain periplasmic proteins in their reductive state (Shouldice et al., 2010). In *B. fragilis,* the TrxB/Trx system is the sole mechanism used to maintain the cellular thiol/disulfide balance and the lack of *trxB* has a major effect on the bacterial growth, oxidative stress response, increased susceptibility to peroxides and thiol oxidants, and survival in intra‐abdominal experimental infections (Reott, Parker, Rocha & Smith, 2009; Rocha et al., 2007). In addition, the TrxB/Trx system seems to be involved in a series of physiological processes in the cytoplasm and periplasm such as the class I aerobic ribonucleotide reductase activity, the protein thiol‐isomerase activities, the periplasm lipoprotein molecular chaperone transport and folding activities (Rocha et al., 2007). Moreover, we cannot rule out at this point of investigation whether the TrxB/Trx redox system may also affect the metal transport activity of the transmembrane hybrid FeoAB fusion system essential for ferrous iron uptake in the *Bacteroides* (Rocha & Smith, 2010; Veeranagouda et al., 2014).

Although we show here that two major *Bacteroides* species within the human colon, *B. vulgatus* and *B. thetaiotaomicron*, can grow on both Fe(III)‐enterobactin and Fe(III)‐salmochelin S4, the characterization of putative TBDT(s) involved in the catechol transport for these species has not been addressed in this study. Nonetheless, we show in supplemental Figure S3 that in the genome of *B. vulgatus* ATCC 8482 contain at least thirteen TBDTs homologs to FepA, CirA, and IroN family of enterobactin and salmochelin S4 transporters in Enterobacteria (Müller, Valdebenito, & Hantke, 2009; Schalk & Guillon, 2013). Therefore, it remains to be determined whether these homologs play any role in *B. vulgatus* utilization of enterobactin and salmochelin S4. Moreover, the absence of significant homologues to periplasmic catechol‐binding protein FepB and ATP‐binding cassette transporter FepCD in the *B. vulgatus* suggests that the cellular compartment transport and removal of iron from catecholate‐type siderophores may also diverge from aerobic and facultative siderophore transport pathway.

The assimilation of iron bound to enterobactin and salmochelin S4 in *Bacteroides* may be highly beneficial to the host because it may counteract and neutralize pathogen strategies to evade host defense mechanisms that limit iron in the intestinal tract. One of these strategies is the ability of enteric pathogens to evade the host mucosal secreted antimicrobial glycoprotein lipocalin‐2 (NGAL). Lipocalin‐2 binds Fe(III)‐enterobactin and iron‐free enterobactin disrupting the bacterial iron supply (Flo et al., 2004; Goetz et al., 2002). To circumvent this host defense mechanism, pathogenic enteric bacteria such as *S. thyphimurium*,* Klebsiella pneumonia*, uropathogenic *E. coli* synthesize salmochelin S4, a dual‐glycosylated enterobactin, to by‐pass the lipocalin‐2 inhibitory effect on enterobactin utilization (Hantke, Nicholson, Rabsch, & Winkelmann, 2003; Müller et al., 2009; Smith, 2007; Valdebenito, Müller, & Hantke, 2007). Thus, the ability of *B. vulgatus* and *B. thetaiotaomicron* to utilize both enterobactin and salmochelin S4 may disrupt enteric bacteria iron supply by virtue of their sheer numbers as they reach 10^11^–10^12^ cfu/g of intestinal content. Again, we think that the differential ability of *Bacteroides* species to utilize xenosiderophores may not only contribute to competition for iron for their own metabolism and growth, but also protection against mass proliferation of pathogenic organisms in the intestinal tract.

In conclusion, this study shows that *Bacteroides* species assimilate Fe(III)‐xenosiderophores for growth under anaerobic conditions in vitro. Despite our limited knowledge of iron bound to xenosiderophores assimilation in anaerobes, our findings support previous studies demonstrating that *Bacteroides* have developed different strategies to deal with the challenges of iron acquisition, genetic regulation and iron‐storage during transitions from anaerobic to aerotolerant metabolism (Betteken, Rocha, & Smith, 2015; Gauss et al., 2012; Rocha & Smith, 2004, 2010, 2013). Moreover, future investigations on the transport and regulatory mechanisms for utilization of catechol siderophores in *B. vulgatus* associated with colitis will advance our understanding on the role iron acquisition systems play in *Bacteroides* pathophysiology.

## Conflict of Interest

None declared.
