# Supplementary material for: Anaerobic utilization of Fe(III)‐xenosiderophores among Bacteroides species and the distinct assimilation of Fe(III)‐ferrichrome by Bacteroides fragilis within the genus
Source: Microbiologyopen. 2017 Apr 11;6(4):e00479. doi: 10.1002/mbo3.479 (PMC5552952; doi:10.1002/mbo3.479)

Supplemental Information

Supplemental Table 1. Utilization of Fe(III)-bound xenosiderophores by *Bacteroides* spp.

Fe(III)-Siderophore / Strains	Enterobactin	Salmochelin	Ferrichrome	Ferrioxamine E	Pyoverdine	Deferrioxamine
B. fragilis NCTC9343	−	−	+	−	−	−
B. fragilis 638R	−	−	+	−	−	−
B. fragilis CLA 267	+	+	+	−	−	−
B. ovatus ATCC 8483	−	−	−	−	−	−
B. thetaiotaomicron VPI 5482	+	+	−	−	−	−
B. vulgatus ATCC 8482	+	+	−	−	−	−
B. vulgatus ATCC 29327	+	+	−	−	−	−
B. vulgauts CLA 341	+	+	−	−	−	−
B. vulgatus 20-15	+	+	−	−	−	−
B. vulgatus 40G2-33	+	+	−	−	−	−
B. vulgatus 10-9	+	+	−	−	−	−
B. vulgatus 16-4	+	+	−	−	−	−

-: No growth; +: growth

Supplemental Table 2 data were collected from experiments performed as described for Fig. 1.

Supplemental Table 2. Relative real time RT-PCR quantification of *B. fragilis fchA1* and *fchA2* mRNAs in defined medium under different growth conditions.

Strains	Growth conditions	Media supplementation	Fold Induction	
			fchA1	*fchA2*
B. fragilis 638R wild-type	iron replete	5 μg/ml PpIX + 100 μM $FeSO_4$	1	1
	iron limiting	5 μg/ml PpIX + 50 μM bipyridyl	0.75	1.4
B. fragilis 638R *ΔfeoAB*	iron replete	5 μg/ml PpIX + 100 μM $FeSO_4$	0.90	1.09
	iron limiting	5 μg/ml PpIX + 50 μM bipyridyl	0.73	1.70
B. fragilis 638R wild-type	heme replete	5 μg/ml hemin + 100 μM $FeSO_4$	1	1
	heme limiting	0.1 μg/ml hemin + 100 μM $FeSO_4$	0.62	0.84
B. fragilis 638R wild-type	iron replete	5 μg/ml PpIX + 20 μM BPS + 10 μM Fe(III)-Fch	1	1
	iron limiting	5 μg/ml PpIX + 20 μM BPS + 0.1 μM Fe(III)-Fch	0.94	0.66

Fold induction of *B. fragilis fchA1* (BF638R_0018) and *fchA2* (BF638R_2503) mRNA expression. Bacteria were grown in defined media containing 5 μg/ml protoporphyrin IX (PpIX) supplemented with 100 μM $FeSO_4$ for iron replete conditions or 50 μM 2,2-bipyridyl for iron limiting conditions as described previously (Rocha and Smith, 2004). Media were also supplemented with 5 μg/ml hemin for heme replete or 0.10 μg/ml hemin for heme limiting conditions as described previously (Rocha et al., 1991). Media containing 5 μg/ml PpIX were also supplemented with 20 μM bathophenanthroline disulfonic acid (BPS) and 10 μM Fe(III)-ferrichrome (Fch) for ferric iron bound siderophore replete condition or 0.1 μM Fe(III)-ferrichrome for ferric iron bound siderophore limiting conditions as described in the materials and methods section. Real-time RT-PCR using the primer sets mentioned below was carried out from total RNA isolated from bacteria grown to mid-log phase in defined media with different supplements as describe above. The 16S rRNA was used as reference to normalize gene expression to a housekeeping gene. The Ct values for *fchA1*and *fchA2* mRNAs were normalized to the Ct of the 16S rRNA and the relative expression of the target genes in iron replete and iron limiting conditions were calculated by applying the $2^{-\Delta\Delta CT}$ method (Livak and Schmittgen, 2001).

List of primers used for real-time PCR in this study.

Primer name	Nucleotide Sequence
BF_0018 FchA1 FOR	CCGGATAGATTCCGATTTCC
BF_0018 FchA1 REV	GGTGAACTTGGGTGTTTTGG
BF_2503 FchA2 FOR	TTGGGCGGTAAACTGATAGG
BF_2503 FchA2 REV	CGGATAGAAATCGCGGTTAG
16S-forward	GATGCGTTCCATTAGGTTGTTG
16S-reverse	CACTGCTGCCTCCCGTAG

References:

Livak KJ, Schmittgen TD. 2001. Analysis of relative gene expression data using real-time quantitative PCR and the $2^{-\Delta\Delta CT}$ method. Methods 25:402-408. PMID: 11846609.

Rocha ER, de Uzeda M, Brock JH. 1991. Effect of ferric and ferrous iron chelators on growth of *Bacteroides fragilis* under anaerobic conditions. FEMS Microbiol Lett 68, 45-50. PMID: 1769555.

Rocha ER, Smith CJ. 2004. Transcriptional regulation of the *Bacteroides fragilis* ferritin gene (*ftnA*) by redox stress. Microbiology 150:2125-2134. PMID: 15256555.

Fig. S1. Multiple alignment of the *B. fragilis* deduced amino acid sequences for FchA1 and FchA2 with *E. coli* FhuA homologue. The loci tags for FchA1 and FchA2 are depicted with strains designations. GenBank accession numbers: BF 638R FchA1 (CBW20634) and FchA2 CBW23011). BF NCTC 9343 FchA1 (CAH05797) and FchA2 (CAH08241). BF YCH46 FchA1 (BAD46768) and FchA2 (BAD49261). *E. coli* W3110 FhuA (BAB96726). Conserved amino acid residues (>50% identity) are labeled with black boxes. Semi-conserved amino acids substitutions are depicted by grey boxes. Alignment of the peptide sequences was performed using vector NTI program AlignX V.11.5.4 with peptide score matrix default data file blosum62mt2 for the comparison of amino acid substitution. The ferrichrome binding residues to *E. coli* FhuA plug region (R81, G99 and Y116) and barrel region (Y244 and Y315) of the mature protein (Locher et al., 1998) are indicated by an asterisk below the consensus sequence. The *E. coli* TonB box consensus sequence (Gudmundsdottir et al., 1989) is depicted by a red bar below the amino acid sequence for indication purpose only.

References:

Locher KP, Rees B, Koebnik R, Mitschler A, Moulinier L, Rosenbusch JP, Moras D.. 1998. Transmembrane signaling across the ligand-gated FhuA receptor: crystal structures of free and ferrichrome-bound states reveal allosteric changes. Cell 95: 771-778. PMID: 9865695.

Gudmundsdottir A, Bell PE, Lundrigan MD, Bradbeer C, Kadner RJ. 1989. Point mutations in a conserved region (TonB box) of *Escherichia coli* outer membrane protein BtuB affect vitamin B12 transport. J Bacteriol. 171:6526-6533. PMID: 2687240.

Fig S1

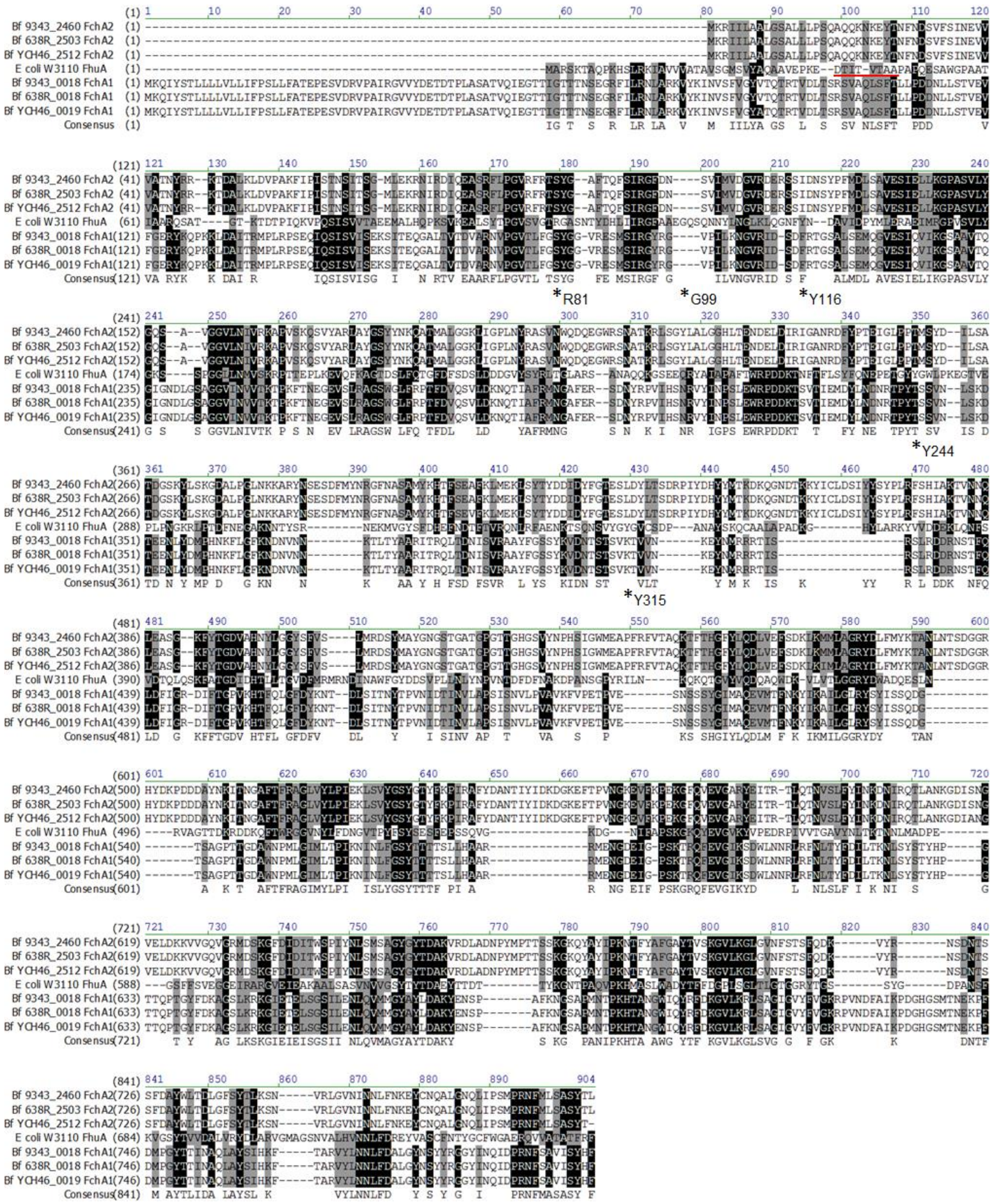
Bf 9343_2460 FchA2
Bf 638R_2503 FchA2
Bf YCH46_2512 FchA2
E coli W3110 FhuA
Bf 9343_0018 FchA1
Bf 638R_0018 FchA1
Bf YCH46_0019 FchA1
Consensus
*R81
*G99
*Y116
*Y244
*Y315

Fig. S2. Phylogenetic relationship of 33 *B. fragilis* 638R predicted TonB-dependent transporters with *E. coli* FhuA homologue (GenBank accession number: (BAB96726). The phylogenetic tree was constructed from multiple amino acid sequences alignment based on ClustalW algorithm in the AlignX program of Vector NTI 11.5.4. The Neighbor Joining method of Saitou and Nei (1987) was used to calculate the distances between all pairs and the calculated relative distance values are depicted in parenthesis following each protein name. The *B. fragilis* 638R TBDT loci tags are labeled for each protein. *E. coli* FhuA, Bf_0018 and Bf_2503 are labeled in bold type style and underlined for clarity.

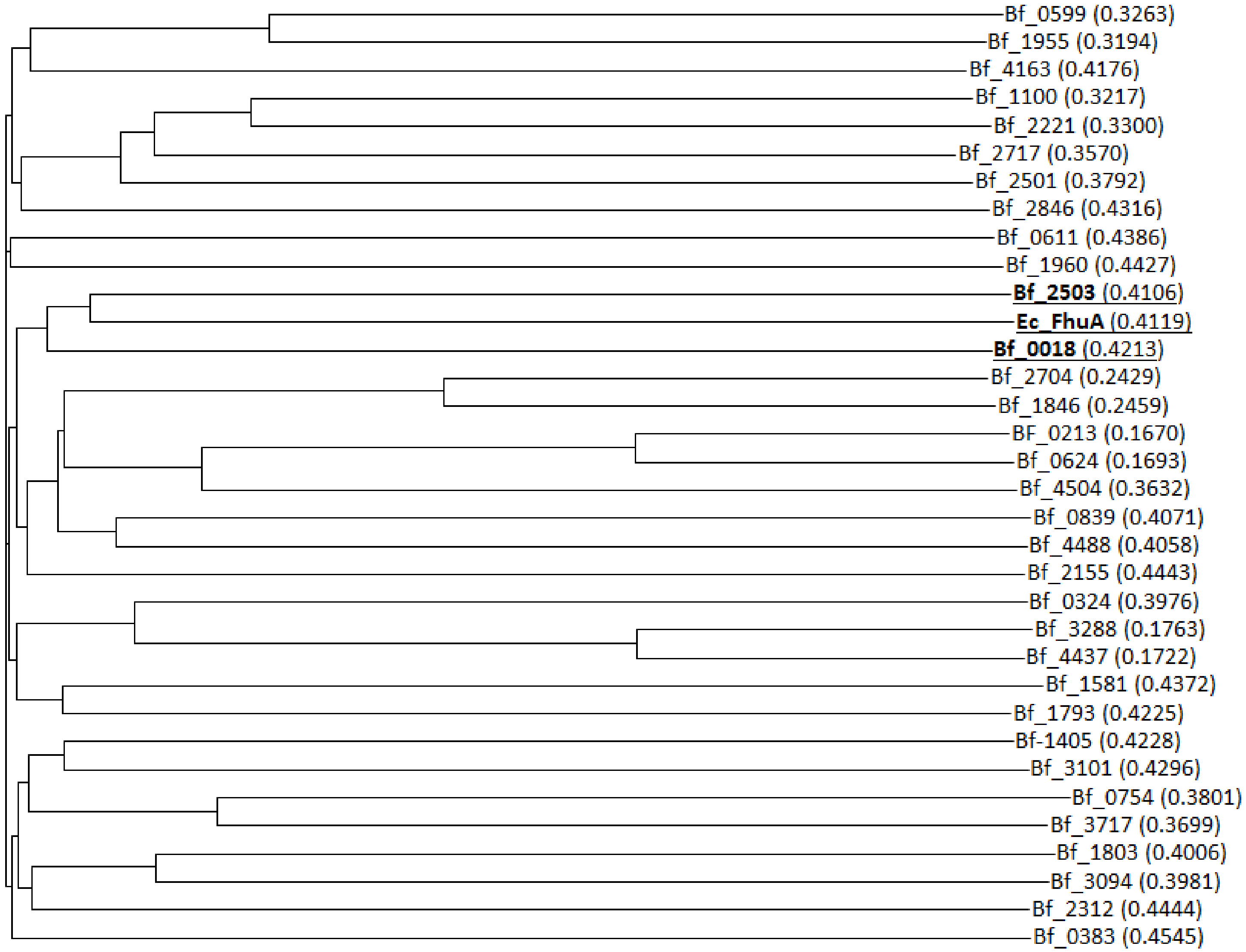

Fig. S3. Phylogenetic relationship of 13 *B. vulgatus* ATCC 8482 (BVU) putative TonB-dependent siderophore transporters with CirA, FepA and IroN homologues representatives from Alpha-, Beta-, and Gamma-proteobacteria. The phylogenetic tree was constructed from multiple amino acid sequences alignment based on ClustalW algorithm in the AlignX program of Vector NTI 11.5.4. The Neighbor Joining method of Saitou and Nei (1987) was used to calculate the distances between all pairs and the calculated relative distance values are depicted in parenthesis following each protein name. GenBank accession numbers: BVU_0851 (ABR38548), BVU_1231 (ABR38921), BVU_1334 (ABR39023), BVU_1487 (ABR39174), BVU_1701 (ABR39377), BVU_2045 (ABR39710), BVU_2193 (ABR39854), BVU_2728 (ABR40378), BVU_3092 (ABR40726), BVU_3095 (ABR40729), BVU_3108 (ABR40742), BVU_3110 (ABR40744) and BVU_4189 (ABR41790). *E. coli* CirA (BAE76632), *E. coli* FepA (BAA35225), *E. coli* IroN (ABD51708), *Neisseria mucosa* FepA (EFV81332), *Rhodospirillum centenum* FepA (ACI97781), *S. enterica* CirA AAL21103), *S. enterica* FepA (AAL19536), *S. enterica* IroN (ADK62215).

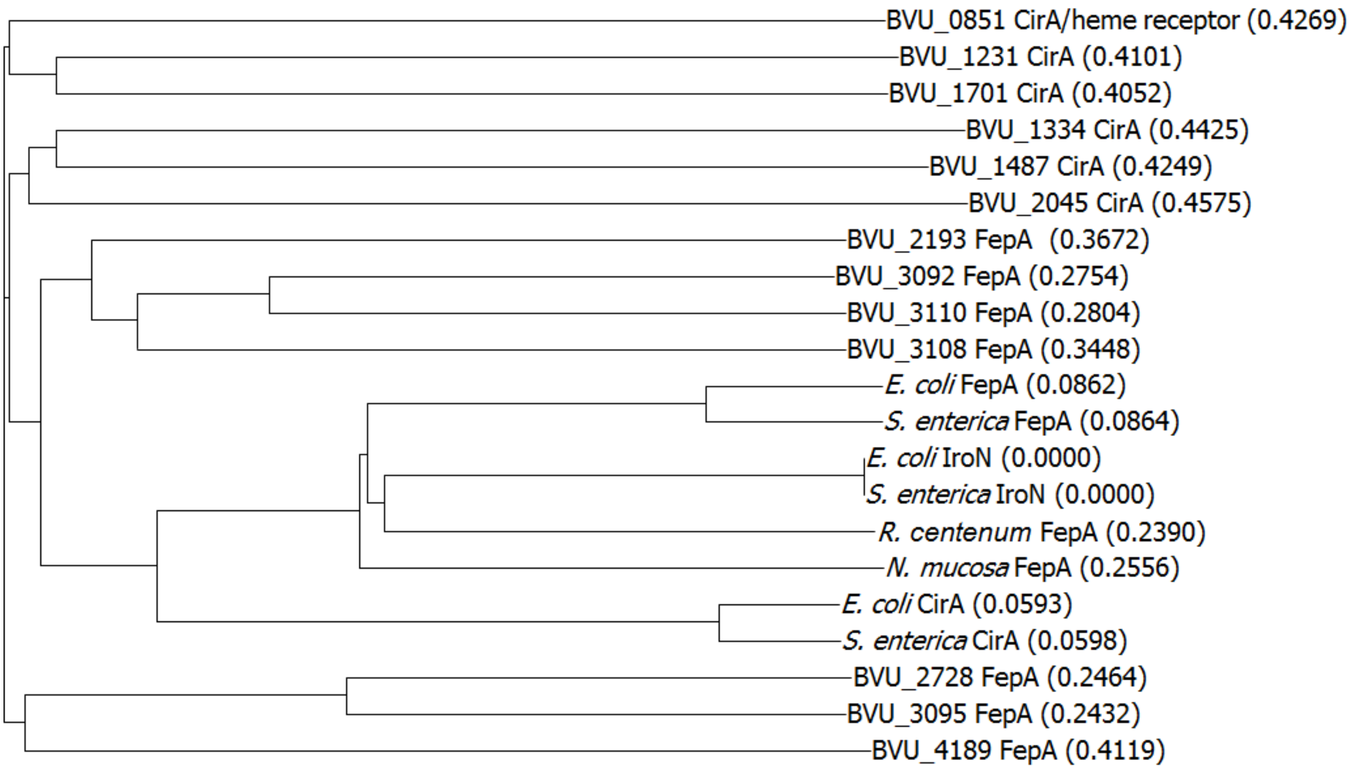

Supplement: Supplementary file 1 [file MBO3-6-na-s001.pdf]
